# Supplementary material for: Hematopoietic reconstitution dynamics of mobilized- and bone marrow-derived human hematopoietic stem cells after gene therapy
Source: Nat Commun. 2023 May 27;14:3068. doi: 10.1038/s41467-023-38448-y (PMC10224916; doi:10.1038/s41467-023-38448-y)
Supplement: Supplementary file 1 — Supplementary Information [file 41467_2023_38448_MOESM1_ESM.pdf]

Hematopoietic reconstitution dynamics of mobilized- and bone marrow-derived human Hematopoietic stem cells after gene therapy

Serena Scala, Francesca Ferrua, Luca Basso-Ricci, Francesca Dionisio, Maryam Omrani, Pamela Quaranta, Raisa Jofra Hernandez, Luca Del Core, Fabrizio Benedicenti, Ilaria Monti, Stefania Giannelli, Federico Fraschetta, Silvia Darin, Elena Albertazzi, Stefania Galimberti, Eugenio Montini, Andrea Calabria, Maria Pia Cicalese, Alessandro Aiuti.

Supplementary items

This file contains:

10 Supplementary Figures + Legends

7 Supplementary Tables

Supplementary Figure 1

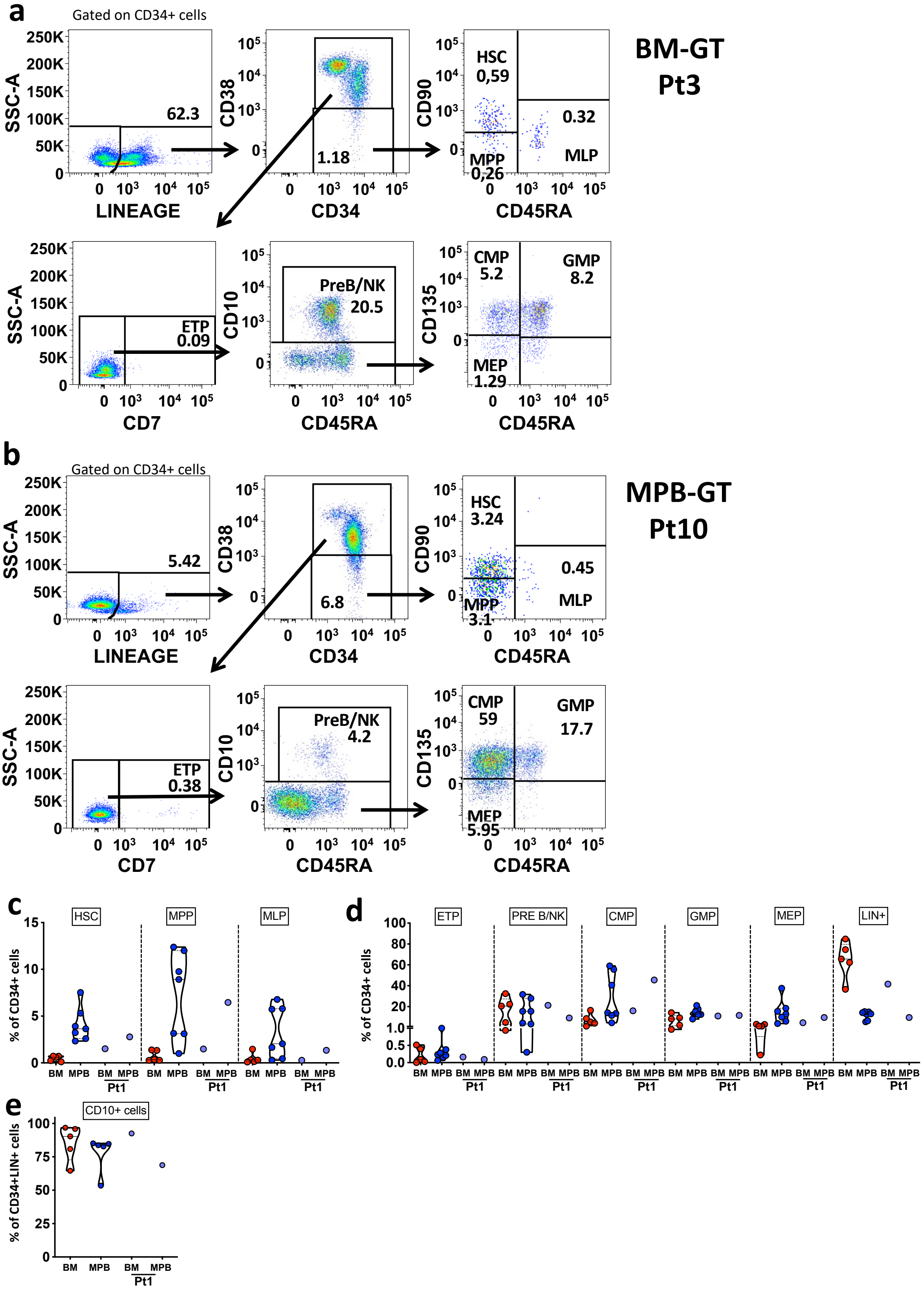

Supplementary figure 1. Representative plots and HSPC composition in BM vs. MPB CD34+ cells. Representative plots of CD34+ composition and gating strategy used for the identification of HSPC subpopulations in (**a**) BM-derived (BM-GT Pt3) or (**b**) MPB-derived (MPB-GT Pt10) CD34+ cells. Numbers within the plots indicate the frequency of each population on total CD34+ cells. (**c**) Frequencies of LIN-CD34+CD38- subpopulations in all the patients analyzed. (**d**) Frequencies of LIN-CD34+CD38+ subpopulations in all the patients analyzed. (**e**) Percentage of CD10+ cells within CD34+LIN+ population in all the patients

analyzed. Lines within the violin plots show the median value, while dashed lines show quartile ranges (BM= Bone Marrow; MPB= Mobilized Peripheral Blood; HSC= Hematopoietic Stem Cells; MPP= Multi-Potent Progenitors; MLP= Multi-lymphoid Progenitors; ETP= Early T cell Progenitors; PreB/NK= B cells and natural killer cell precursors; CMP= Common Myeloid Progenitors; GMP= Granulocytes-Monocytes Progenitors; MEP= Megakaryocytes Erythrocytes Progenitors). Source data are provided as a Source Data file.

Supplementary Figure 2

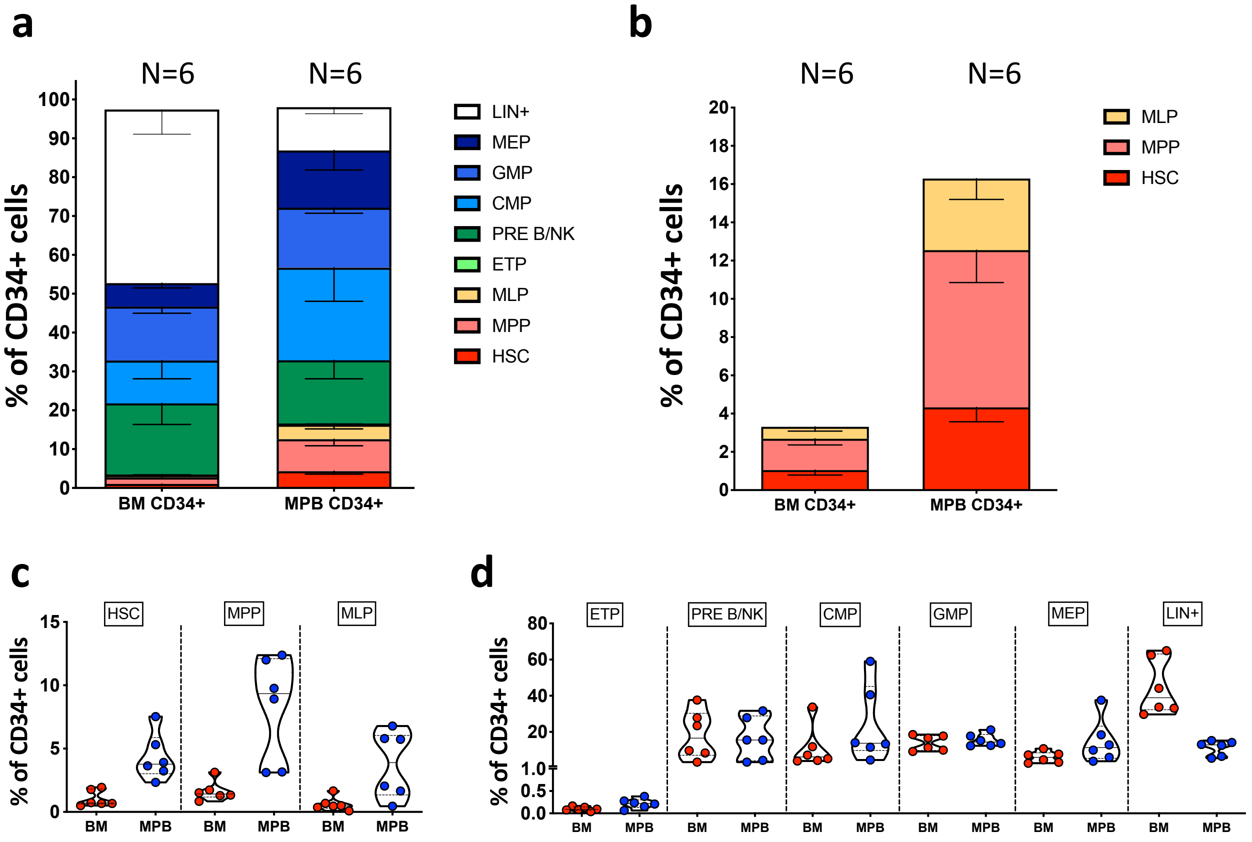

Supplementary Figure 2. HSPC composition in BM vs. MPB CD34+ cells derived from the same patients. (**a**) CD34+ cell composition in CD34+ cells isolated from BM (n=6 biologically independent samples) or MPB (n=6 biologically independent samples) samples of the same patients. BM composition was performed before mobilization procedures. We did not analyze the BM sample of one patient (Pt9) before mobilization and his MPB composition was not included in this analysis. (**b**) Focus on CD34+LIN-CD38- cell compartment in CD34+ cells isolated from BM or MPB samples of the same patients. (**c**) Frequencies of LIN-CD34+CD38- subpopulations in CD34+ cells isolated from BM or MPB samples of the same patients. (**d**) Frequencies of LIN-CD34+CD38+ subpopulations in CD34+ cells isolated from BM or MPB samples of the same patients.

For all the graphs in the figure, data in the stacked bar graphs are shown as Mean+/- Standard Error Mean, while, in the violin plots representation, lines within the violin show the median value, while dashed lines show quartile ranges (BM= Bone Marrow; MPB= Mobilized Peripheral Blood; HSC= Hematopoietic Stem Cells; MPP= Multi-Potent Progenitors; MLP= Multi-lymphoid Progenitors; ETP= Early T cell Progenitors; PreB/NK= B cells and natural killer cell precursors; CMP= Common Myeloid Progenitors; GMP= Granulocytes-Monocytes Progenitors; MEP= Megakaryocytes Erythrocytes Progenitors; LIN=lineage). Source data are provided as a Source Data file

Supplementary Figure 3

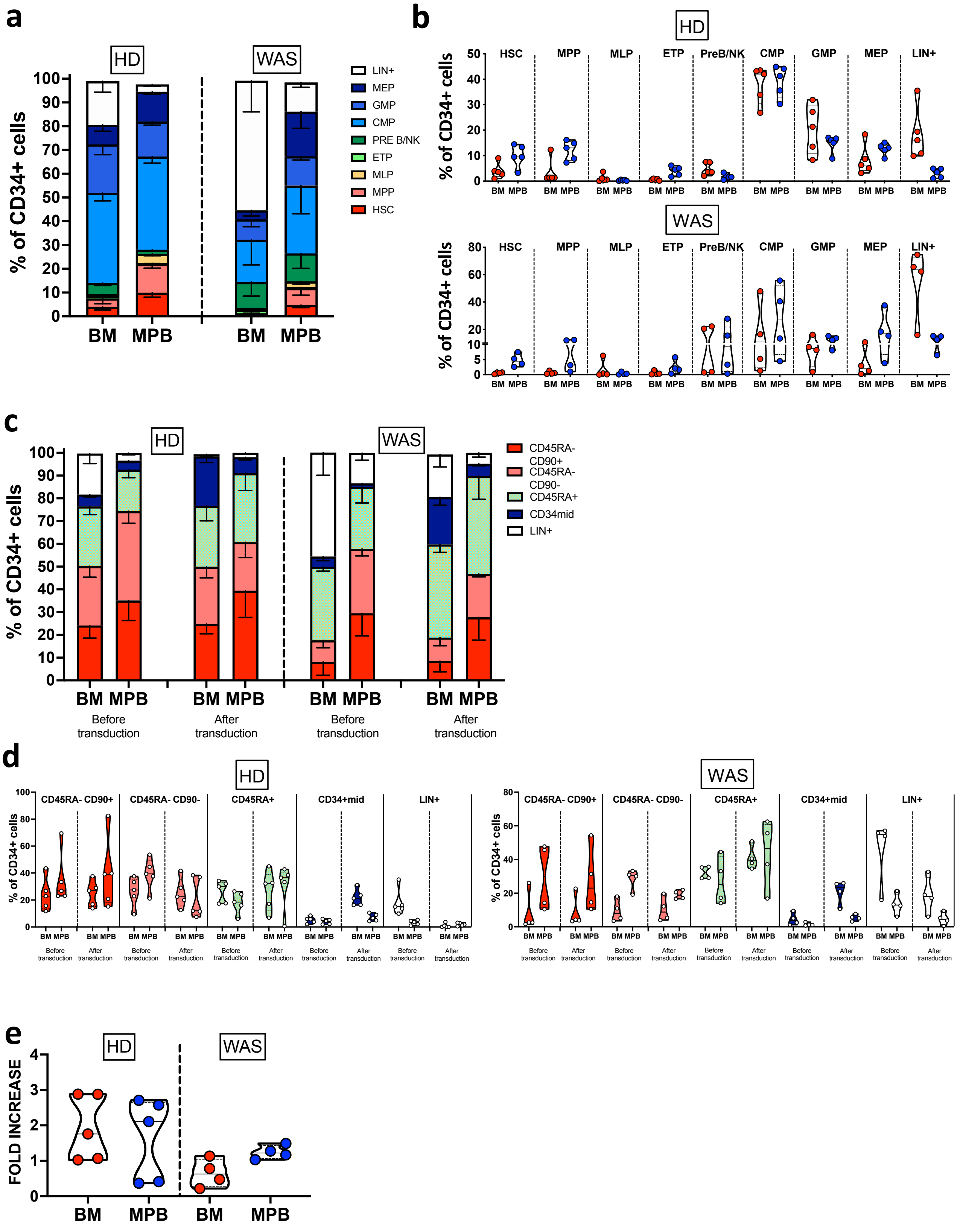

Supplementary Figure 3. Composition of HD and WAS patients' CD34+ cells from BM and MPB sources is maintained after transduction. (**a**) Bar plots showing the CD34+ cell composition with the conventional gating strategy (with the use of CD38 marker) of HD (n=5 biologically independent samples) and WAS (n=4 biologically independent samples) patients' BM and MPB samples before *in vitro* transduction. (**b**) Violin showing the CD34+ cell

composition with the conventional gating strategy of (top) HD and (bottom) WAS patients' BM and MPB samples before *in vitro* transduction. (**c**) Bar plots showing the CD34+ cell composition with the alternative gating strategy (without the use of CD38 marker) of the samples undergoing *in vitro* transduction (n=5 biologically independent samples for HD and n=4 biologically independent samples for WAS patients). (**d**) Violin plots showing the CD34+ cell composition with the alternative gating strategy of the HD (left) and WAS (right) samples undergoing *in vitro* transduction. (**e**) Fold increase of the cell count after transduction on the number of seeded cells for BM and MPB CD34+ cells from HD and WAS patients.
For all the graphs in the figure, data in the stacked bar graphs are shown as Mean+/- Standard Error Mean, while, in the violin plots representation, lines within the violin show the median value, while dashed lines show quartile ranges (HD= Healthy donors, WAS= Wiskott-Aldrich Syndrome; BM= Bone Marrow; MPB= Mobilized Peripheral Blood; HSC= Hematopoietic Stem Cells; MPP= Multi-Potent Progenitors; MLP= Multi-lymphoid Progenitors; ETP= Early T cell Progenitors; PreB/NK= B cells and natural killer cell precursors; CMP= Common Myeloid Progenitors; GMP= Granulocytes-Monocytes Progenitors; MEP= Megakaryocytes Erythrocytes Progenitors). Source data are provided as a Source Data file

Supplementary Figure 4

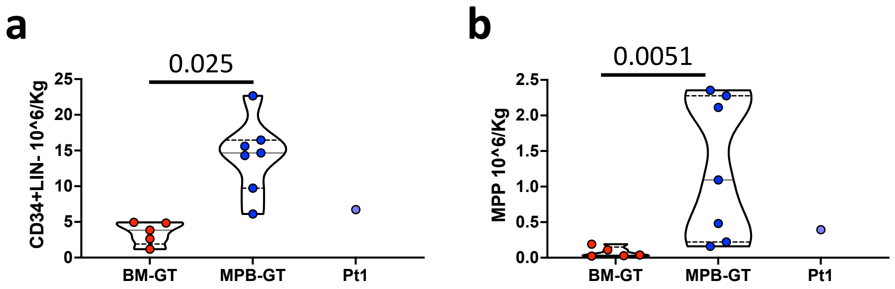

Supplementary Figure 4. Amount of CD34+LIN- cells and MPP infused in BM-GT and MPB-GT patients. (**a-b**) Estimation of the number of infused (**a**) CD34+LIN- cells (HSPC) and (**b**) MPP in BM-GT, MPB-GT and Pt1. For Pt1 we reported the overall amount of cells from both sources. Lines within the violin show the median value, while dashed lines show quartile ranges (Statistical test for groups' comparisons: Two-sided Mann-Whitney; only statistically significant values are reported within the graphs; HSPC= Hematopoietic Stem/Progenitor Cells; MPP= Multi-Potent Progenitors). Source data are provided as a Source Data file

Supplementary Figure 5

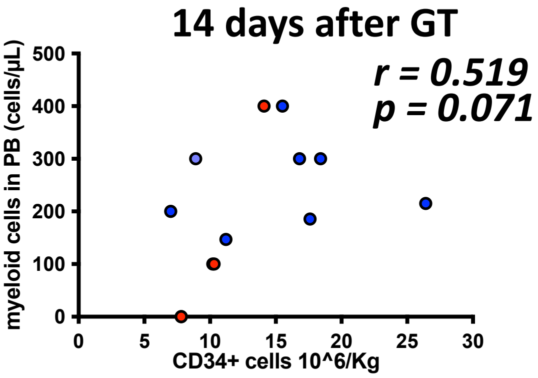

Supplementary Figure 5. Correlation between CD34+ cell dose and early myeloid reconstitution. Dot plots showing the correlation between CD34+ cell dose with myeloid cell counts at 14 days after GT. Blue dots: MPB-GT patients (n=7); Red dots: BM-GT patients (n=5); Purple dot: Pt1 (Statistical test: Two-sided Spearman r test; PB =Peripheral Blood).

Supplementary Figure 6

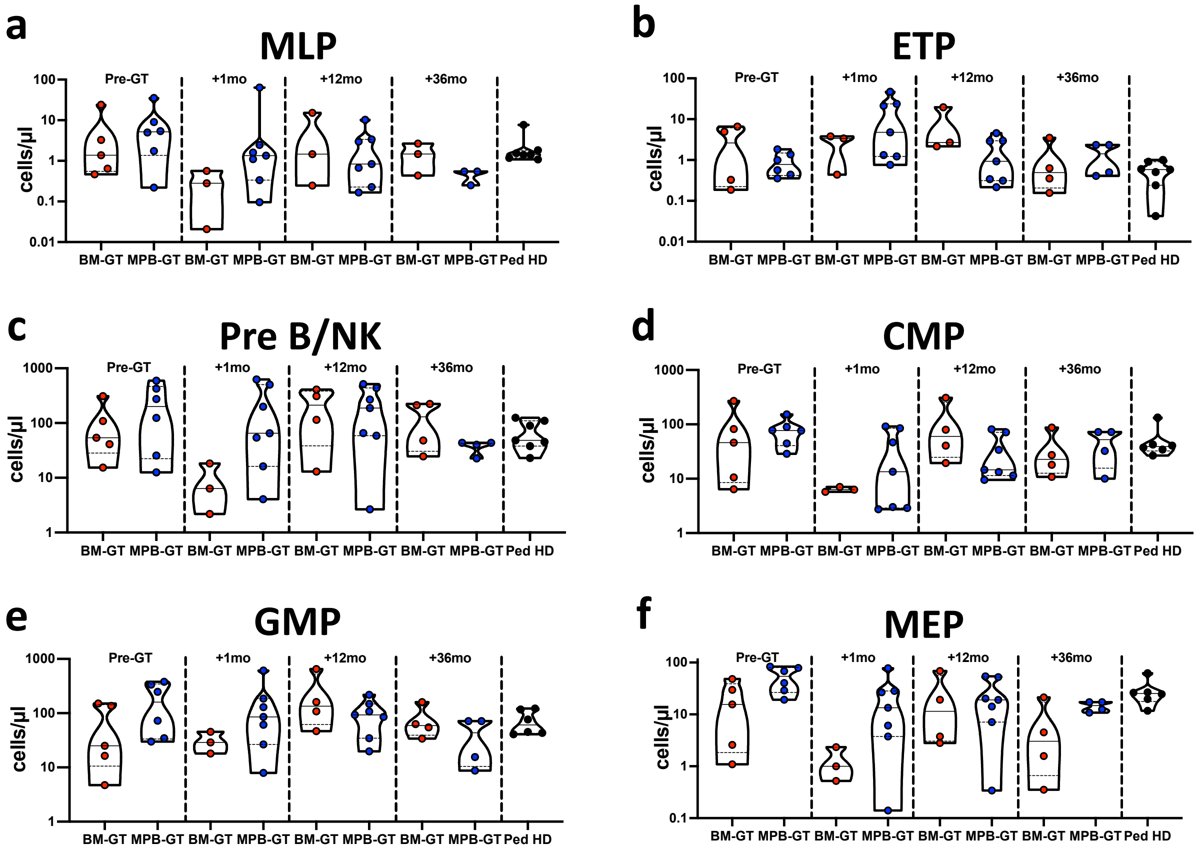

Supplementary Figure 6. Absolute count of HSPC subpopulations after GT in BM-GT and MPB-GT patients up to 3 years after transplant. Violin plots showing (**a**) MLP, (**b**) ETP, (**c**) PreB/NK, (**d**) CMP, (**e**) GMP and (**f**) MEP cell counts at different time points after GT in BM-GT (red dots) and MPB-GT(blue dots). Black dots display values for Ped HD. Lines within the violin show the median value, while dashed lines show quartile ranges. (BM-GT= Bone Marrow gene therapy patients; MPB-GT= Mobilized Peripheral Blood gene therapy patients; Ped HD= Pediatric Healthy Donors; HSC= Hematopoietic Stem Cells; MPP= Multi-Potent Progenitors; MLP= Multi-lymphoid Progenitors; ETP= Early T cell Progenitors; PreB/NK= B cells and natural killer cell precursors; CMP= Common Myeloid Progenitors; GMP= Granulocytes-Monocytes Progenitors; MEP= Megakaryocytes Erythrocytes Progenitors). Source data are provided as a Source Data file.

Supplementary Figure 7

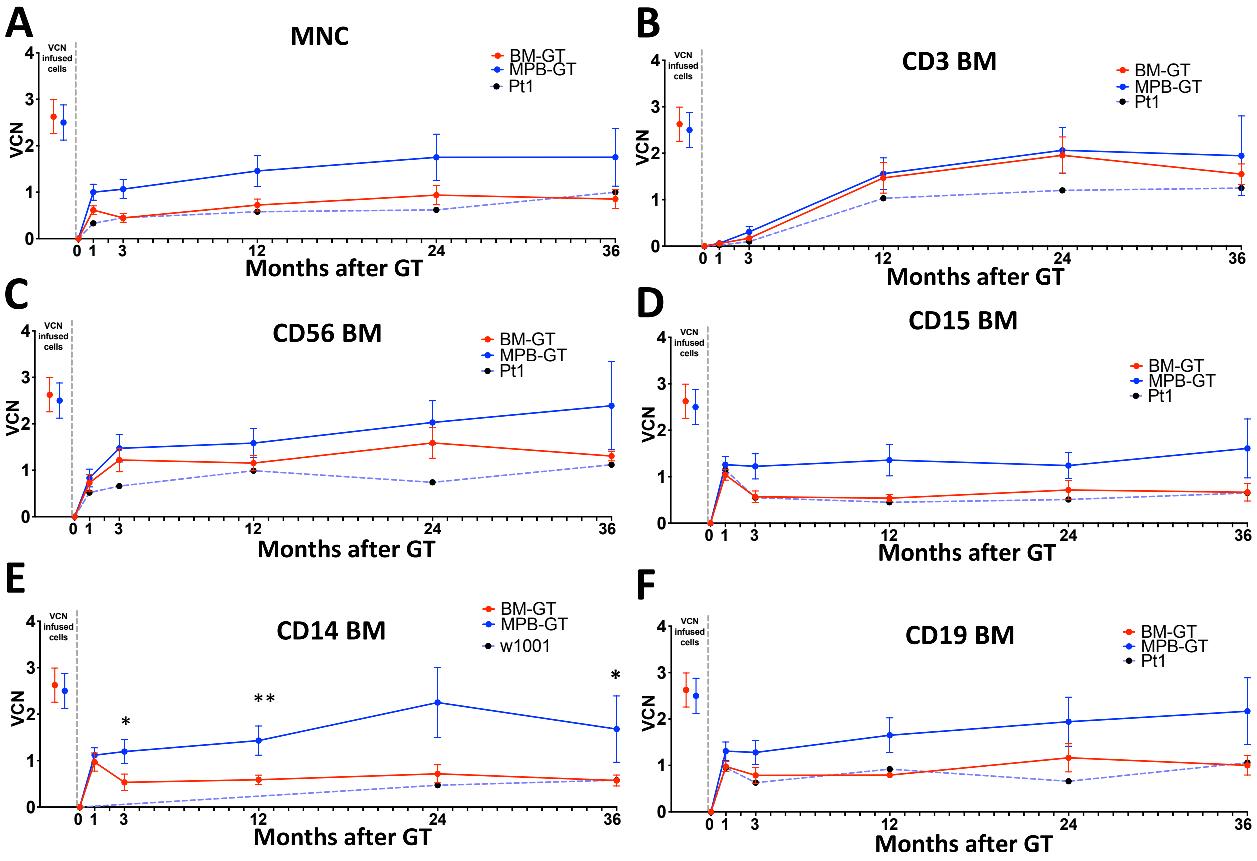

Supplementary Figure 7. Gene correction of BM progenitors in BM-GT and MPB-GT patients. All data are shown as mean +/- SEM. Vector copy number (VCN) measurement up to 3 years after gene therapy (GT) in (**a**) Mononuclear cells (MNC) and purified (**b**) CD3 BM, (**c**) CD56 BM, (**d**) CD15 BM, (**e**) CD14 BM and (**f**) CD19 BM in BM-GT(n=5 biologically independent samples), MPB-GT (n=7 biologically independent samples) and Pt1. (Statistical tests on VCN collected over time: two-sided tests from longitudinal models on CD34+ cell dose, no adjustment for multiple comparisons were made *=p<0.05; **=p<0.01; exact p values were reported in Supplementary Table 1; BM= Bone Marrow; BM-GT= Bone Marrow gene therapy patients; MPB-GT= Mobilized Peripheral Blood gene therapy patients).

Supplementary Figure 8

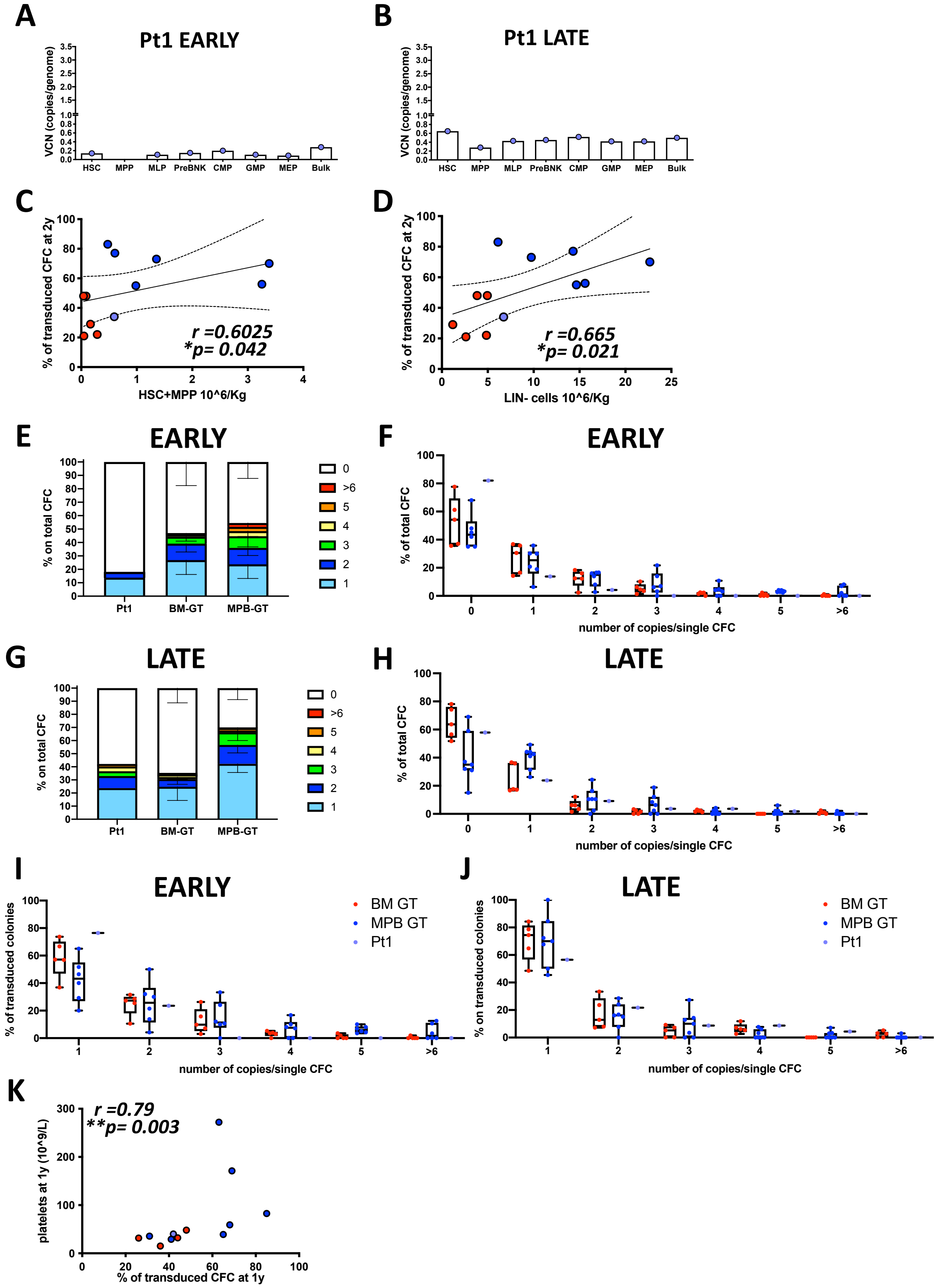

Supplementary Figure 8. VCN distribution in single colonies from BM-GT and MPB-GT patients at early and late phases after transplant. (**a-b**)Estimation of vector copy number (VCN) in sorted HSPC subpopulations in Pt1 at early(90 days) and late(>1 year) time points after GT. (**c-d**)Dot plots showing the correlation between the number of (**c**) HSC+MPP or (**d**) LIN- cells infused in WAS-GT patients with the percentage of transduced Colony Forming Cells

(CFC) at 2 years after GT. (**e-h**)Bar Graphs displaying the distribution of the number of integrated copies/single colony of therapeutic vector on the total CFC analyzed. CFC were derived from CD34+ cells purified from BM of GT patients at (**e-f**) early(30 days, n=5 biologically independent samples for BM-GT and n=6 biologically independent samples for MPB-GT) and (**g-h**) late(>1 year, n=5 biologically independent samples for BM-GT and n=5 biologically independent samples for MPB-GT) time points after GT. (**i-j**) Distribution of the number of copies/single colonies of therapeutic vector on positive CFC analyzed at (**i**) early (n=5 biologically independent samples for BM-GT and n=6 biologically independent samples for MPB-GT) and (**j**) late (n=5 biologically independent samples for BM-GT and n=6 biologically independent samples for MPB-GT) phases after GT. (**k**) Dot plot showing the correlation between the percentage of transduced CFC and the number of platelets at 1 year after GT. Exact p values are shown. Blue dots: MPB-GT patients (n=7); Red dots: BM-GT patients (n=4); Purple dot: Pt1 (H-I)

For all the graphs in the figure, data in the stacked bar graphs are shown as Mean+/- Standard Error Mean, while, in the box and whiskers plots representation, boxes extend from the 25th to 75th percentiles (line in the middle indicate the median value) and the whiskers mark the minimum and the maximum values. (Statistical test for correlation: Two-sided Spearman r test; Statistical test for groups' comparison: Two-sided Mann-Whitney; HSC= Hematopoietic Stem Cells; MPP= Multi-Potent Progenitors; MLP= Multi-lymphoid Progenitors; ETP= Early T cell Progenitors; PreB/NK= B cells and natural killer cell precursors; CMP= Common Myeloid Progenitors; GMP= Granulocytes-Monocytes Progenitors; MEP= Megakaryocytes Erythrocytes Progenitors; BM-GT= Bone Marrow gene therapy patients; MPB-GT: Mobilized Peripheral Blood gene therapy patients). Source data for panel A and B are provided as a Source Data file.

Supplementary Figure 9

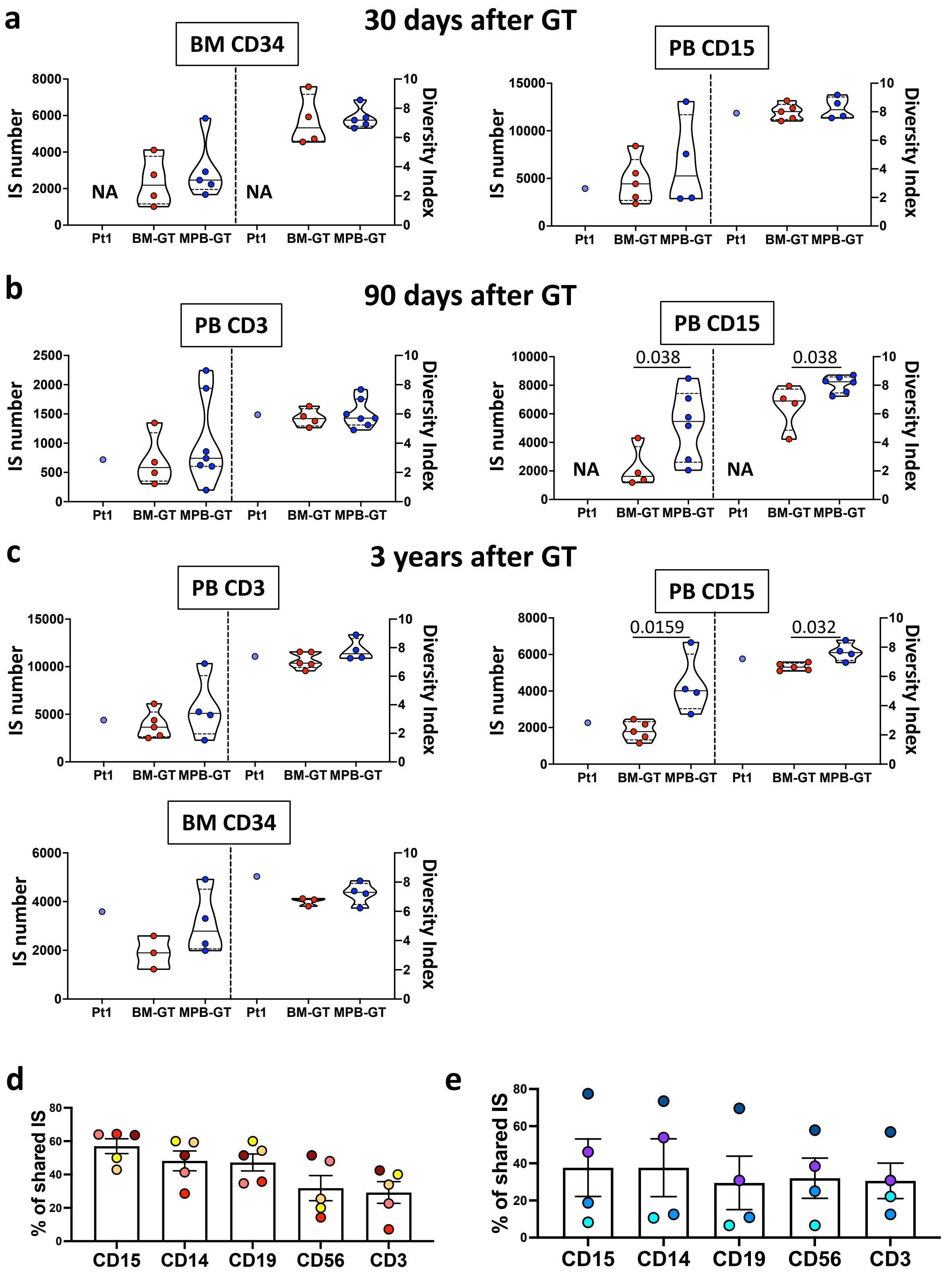

Supplementary Figure 9. Integration site number and diversity index in Myeloid, Lymphoid and Stem cell compartments in MPB-GT and BM-GT patients. Violin plots showing the total number of Integration Sites (IS) and the relative Diversity index of purified CD15+, CD3+ and CD34+ cells from WAS Pt1, BM-GT and MPB-GT patients at: (**a**) 30 days, (**b**) 90 days and (**c**) 3 years after GT. IS numbers have been normalized on the basis of the total

amount of DNA used for retrieving IS. NA= not available. (**d-e**) Frequency of IS retrieved from sorted HSC+MPP populations shared with sorted mature PB populations at steady state (>1 year post GT) in BM-GT (left, n=5 biological replicates) or MPB-GT (right, n=4 biological replicates) patients. Dots with the same color in all the subsets show the IS sharing levels for the same patient.

For all the graphs in the figure, data in the stacked bar graphs are shown as Mean+/- Standard Error Mean, while, in the violin plots representation, lines within the violin show the median value, while dashed lines show quartile ranges (Statistical test for groups' comparisons: Two-sided Mann-Whitney; exact p values are shown within the graphs. BM-GT= Bone Marrow gene therapy patients; MPB-GT: Mobilized Peripheral Blood gene therapy patients; BM = Bone Marrow; PB= Peripheral Blood). Source data are provided as a Source Data file

Supplementary Figure 10

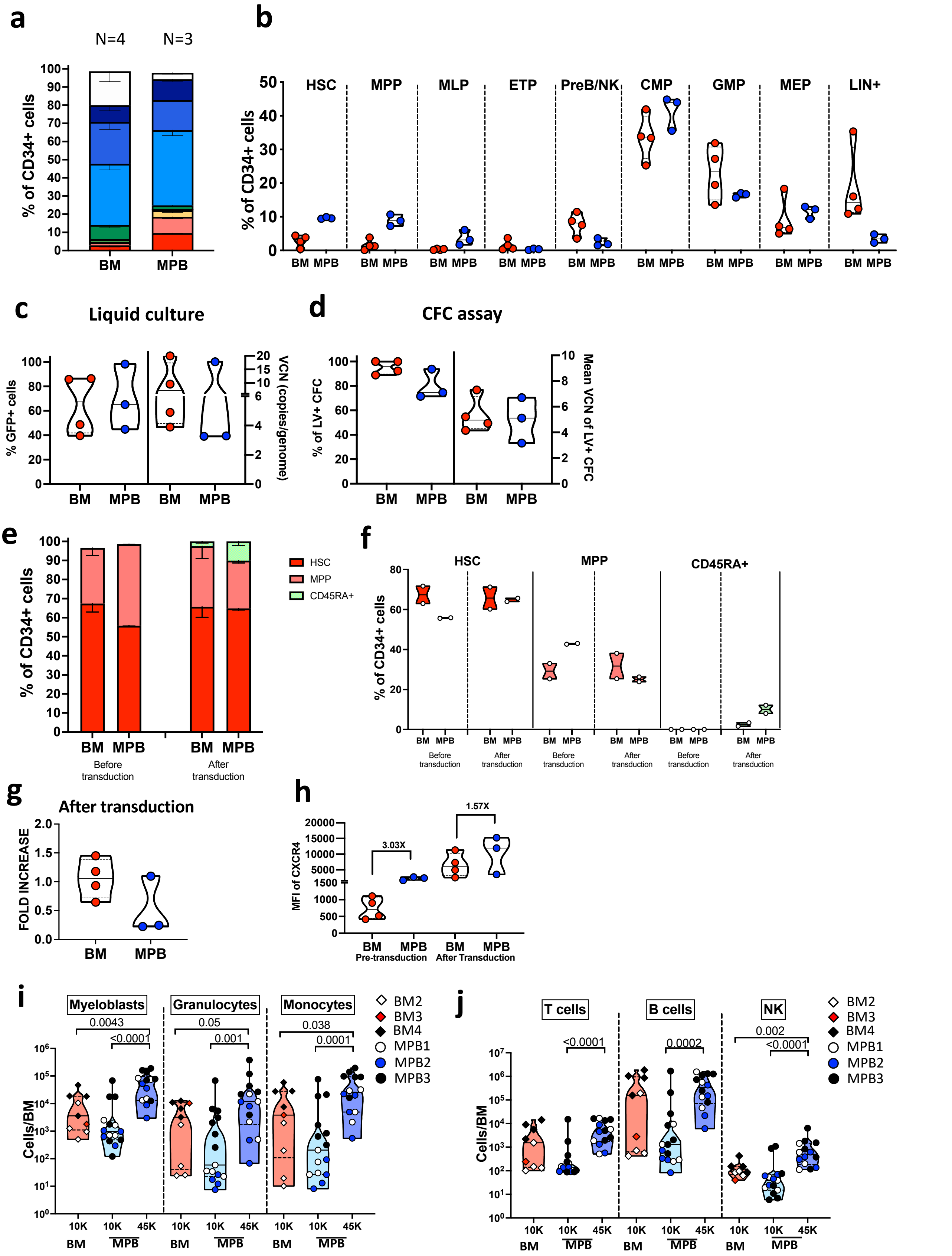

Supplementary Figure 10. Characteristics of BM or MPB primitive subsets after transduction and their *in vivo* output. (**a-b**) Cell composition of healthy donor(HD) BM (n=4 biological replicates) or MPB (n=3 biological replicates) CD34+ cells before sorting of HSC+MPP

populations for *in vitro* and *in vivo* assays. (**c**) Percentage of GFP+ cells and VCN of transduced HSC+MPP sorted from BM (n=4) or MPB (n=3) CD34+ cells after 15 days of liquid culture. (**d**) Percentage of LV+ colonies and mean VCN of CFC derived from transduced HSC+MPP sorted from BM (n=4) or MPB (n=3) CD34+ cells after 15 days of CFC assay. (**e-f**) Composition of sorted HSC+MPP from BM (n=2 biological replicates) or MPB (n=2 biological replicates) before and after culture exploiting alternative gating strategy proposed in Supplementary Fig. 3. (**g**) Fold increase of the cell count after transduction on the number of seeded cells of primitive HSC+MPP populations isolated from BM or MPB CD34+ cells. (**h**) MFI of CXCR4 expressed on HSC+MPP populations before and after transduction. Fold of the MFI in MPB vs. BM HSC+MPP populations is reported. (**i-j**) Absolute count of human myeloid (**i**) and lymphoid (**j**) subpopulations in the BM of 10k BM, 10k MPB and 45k MPB mice 12 weeks after transplantation.
For all the graphs in the figure, data in the stacked bar graphs are shown as Mean+/- Standard Error Mean, while, in the violin plots representation, lines within the violin show the median value, while dashed lines show quartile ranges (Statistical test for groups' comparisons: Two-sided Mann-Whitney. Only p values <0.05 are presented within the graphs; BM= Bone Marrow; MPB=Mobilized Peripheral Blood; GFP= Green Fluorescent Protein; VCN=Vector Copy Number; LV = Lentiviral Vector; CFC= Colony Forming Cell; MFI=mean fluorescent intensity; NK= Natural Killer). Source data are provided as a Source Data file.

Supplementary Table 1

		Months after GT						
	Subset	1	2	3	6	12	24	36
PB	CD15	0.1561	**0.0261**	**0.02**	**0.0098**	**0.0255**	**0.0236**	**0.0102**
	CD14	0.2454	**0.009**	**0.0143**	**0.0052**	**0.0271**	**0.0175**	**0.0052**
	CD19	0.6099	0.0669	**0.0154**	**0.0208**	0.0524	**0.0428**	0.1137
	CD3	0.8853	0.6985	0.4692	0.9927	0.4508	0.863	0.438
	CD56	0.4447	0.1073	0.3322	0.1611	0.3307	0.2713	0.042
BM	CD15	0.7797	ND	0.2103	ND	0.2563	0.5175	0.3348
	CD14	0.0684	ND	**0.014**	ND	**0.004**	0.0725	**0.0393**
	CD19	0.2759	ND	0.2068	ND	0.1013	0.4567	0.1831
	CD3	NA	ND	NA	ND	NA	NA	NA
	CD56	NA	ND	NA	ND	NA	NA	NA
	CD34	0.2062	ND	0.0822	ND	0.1365	0.1999	0.1573

Supplementary Table 1. Exact p values of longitudinal statistical models normalized on CD34+ cell dose to compare VCN in distinct hematopoietic lineages at different time points after infusion of gene-corrected cells in BM-GT vs. MPB-GT groups. Values reported in bold are statistically significant. NA= not applicable; statistical test for source*time effect was not significant. ND: not done; BM aspirates were not performed at 2 and 6 months after GT. (PB= Peripheral blood; BM= Bone Marrow).

Supplementary Table 2

		Subset	1 month	3 months	12 months	36 months
PT1	BM	MNC	3538	N.A	2589	3083
		CD34	N.A	N.A	N.A	3585
	PB	MNC	5032	N.A	2041	3810
		CD15	3934	N.A	N.A	2265
		CD3	N.A	718	N.A	4388
PT2	BM	MNC	1720	N.A	2063	2037
		CD34	1013	N.A	N.A	1223
	PB	MNC	1401	N.A	N.A	3505
		CD15	3043	1865	N.A	1506
		CD3	N.A	493	N.A	2772
PT3	BM	MNC	2248	N.A	2691	3531
		CD34	2759	N.A	N.A	N.A
	PB	MNC	1755	N.A	4042	3848
		CD15	5546	1190	N.A	2181
		CD3	N.A	675	N.A	3641
PT4	BM	MNC	1188	N.A	1569	924
		CD34	N.A	N.A	N.A	N.A
	PB	MNC	1094	N.A	2966	1742
		CD15	2336	1378	N.A	1147
		CD3	N.A	305	N.A	2513
PT6	BM	MNC	2877	N.A	3422	3699
		CD34	4112	N.A	N.A	2591
	PB	MNC	2816	N.A	6857	3984
		CD15	8409	4303	N.A	2458
		CD3	N.A	1346	N.A	6097
PT7	BM	MNC	2303	N.A	2933	2239
		CD34	1615	N.A	N.A	1895
	PB	MNC	2288	N.A	3520	3452
		CD15	4430	N.A	N.A	1779
		CD3	N.A	N.A	N.A	4369
PT8	BM	MNC	5694	N.A	N.A	3603
		CD34	N.A	N.A	N.A	2271
	PB	MNC	3714	N.A	8931	7793
		CD15	2960	N.A	N.A	4108
		CD3	N.A	604	N.A	5258
PT9	BM	MNC	8603	N.A	5770	9666
		CD34	N.A	N.A	N.A	4911
	PB	MNC	3120	N.A	15593	8839
		CD15	N.A	7079	N.A	6657
		CD3	N.A	741	N.A	10327
PT10	BM	MNC	1124	N.A	2509	2314
		CD34	2237	N.A	N.A	1987
	PB	MNC	N.A	N.A	2164	3248
		CD15	2877	2796	N.A	2735
		CD3	N.A	198	N.A	2277
PT11	BM	MNC	2307	N.A	4846	4908
		CD34	2468	N.A	N.A	3305
	PB	MNC	1187	N.A	1749	5631
		CD15	N.A	5165	N.A	3921
		CD3	N.A	2243	N.A	4924
PT12	BM	MNC	3789	N.A	5710	7261
		CD34	5849	N.A	N.A	N.A
	PB	MNC	6363	N.A	6895	7923
		CD15	13067	8471	N.A	N.A
		CD3	N.A	1938	N.A	N.A
PT13	BM	MNC	6044	N.A	4229	N.A
		CD34	2926	N.A	N.A	N.A
	PB	MNC	2550	N.A	5740	N.A
		CD15	7568	5759	N.A	N.A
		CD3	N.A	624	N.A	N.A
PT14	BM	MNC	2317	N.A	N.A	N.A
		CD34	1677	N.A	N.A	N.A
	PB	MNC	1942	N.A	N.A	N.A
		CD15	7568	2048	N.A	N.A
		CD3	N.A	857	N.A	N.A

Supplementary Table 2. Integration site number retrieved from each WAS-GT patient and each lineage to perform comparison of IS number between BM-GT and MPB-GT group. Number

of IS reported are normalized according to the amount of DNA used for IS retrieval (See also Methods Section.) (PB= Peripheral Blood; BM= Bone Marrow; MNC= Mononuclear Cells; N.A. = Not available).

Supplementary Table 3

Model		AIC
**1**	**log(h) = (I) + DNA + VCN + mrk + t + source + mrk * t**	**-96,59**
2	log(h) = (I) + DNA + VCN + mrk + t + source + mrk * t + source * t	-94,87
3	log(h) = (I) + DNA + VCN + mrk + t + source + mrk * source + mrk * t	-94,22
4	log(h) = (I) + DNA + VCN + mrk + t + source + mrk * source + mrk * t + source * t	-92,34
5	log(h) = (I) + DNA + VCN + mrk + t + source + mrk * source + mrk * t + source * t + mrk * source * t	-89,23
6	log(h) = (I) + DNA + VCN + mrk + source	-85,23
7	log(h) = (I) + DNA + VCN + mrk + t + mrk * t	-85,2
8	log(h) = (I) + DNA + VCN + mrk + source + mrk * source	-84,48
9	log(h) = (I) + DNA + VCN + mrk + t + source	-84,36
10	log(h) = (I) + DNA + VCN + mrk + t + source + mrk * source	-83,64
11	log(h) = (I) + DNA + VCN + mrk + t + source + source * t	-82,76
12	log(h) = (I) + DNA + VCN + mrk + t + source + mrk * source + source * t	-81,8
13	log(h) = (I) + DNA + VCN + mrk	-79,19
14	log(h) = (I) + DNA + VCN + source	-77,95
15	log(h) = (I) + DNA + VCN + mrk + t	-77,33
16	log(h) = (I) + DNA + VCN + t + source	-76,08
17	log(h) = (I) + DNA + VCN + t + source + source * t	-74,43
18	log(h) = (I) + DNA + VCN	-73,73
19	log(h) = (I) + DNA + VCN + t	-71,76

Supplementary Table 3. Linear models for comparing the behavior of the logarithmic dataset entropy (*h*), each featuring different combinations of variable interactions ("*I*": intercept; "*DNA*": amount of DNA used to retrieve IS; "*VCN*": Vector copy number; "*mrk*": cell population; "*t*": time point; "*source*": HSPC source for GT). The models were ranked according to the AIC score (from the lowest to the highest), in bold the best model according to the lowest AIC. (AIC= Akaike Information Criterion)

Supplementary Table 4

Model		AIC
**1**	**log(nIS) = (I) + DNA + VCN + mrk + t + source + mrk * t**	**262,83**
2	log(nIS) = (I) + DNA + VCN + mrk + t + source + mrk * t + source * t	263,74
3	log(nIS) = (I) + DNA + VCN + mrk + t + source + mrk * source + mrk * t	264,84
4	log(nIS) = (I) + DNA + VCN + mrk + t + source + mrk * source + mrk * t + source * t	266,02
5	log(nIS) = (I) + DNA + VCN + mrk + t + source + mrk * source + mrk * t + source * t + mrk * source * t	269,73
6	log(nIS) = (I) + DNA + VCN + mrk + t + mrk * t	270,02
7	log(nIS) = (I) + DNA + VCN + mrk + t + source	283,1
8	log(nIS) = (I) + DNA + VCN + mrk + t + source + mrk * source	283,44
9	log(nIS) = (I) + DNA + VCN + mrk + source	283,58
10	log(nIS) = (I) + DNA + VCN + mrk + t + source + source * t	283,84
11	log(nIS) = (I) + DNA + VCN + mrk + source + mrk * source	283,94
12	log(nIS) = (I) + DNA + VCN + mrk + t + source + mrk * source + source * t	284,58
13	log(nIS) = (I) + DNA + VCN + mrk	285,12
14	log(nIS) = (I) + DNA + VCN + mrk + t	285,93
15	log(nIS) = (I) + DNA + VCN + source	298,54
16	log(nIS) = (I) + DNA + VCN	298,69
17	log(nIS) = (I) + DNA + VCN + t + source	300,13
18	log(nIS) = (I) + DNA + VCN + t	300,6
19	log(nIS) = (I) + DNA + VCN + t + source + source * t	301,07

Supplementary Table 4. Linear models for comparing the behavior of the logarithmic number of IS in the dataset, each featuring different combinations of variable interactions ("I": intercept; "DNA": amount of DNA used to retrieve IS; "VCN": Vector copy number; "mrk": cell population; "t": time point; "source": HSPC source for GT). The models were ranked according to the AIC score (from the lowest to the highest), in bold the best model according to the lowest AIC. (IS= Integration Site; AIC=Akaike Information Criterion)

Supplementary Table 5

		EARLY			LATE		
		IS in PB Myeloid compartment			IS in PB Myeloid compartment		
Source	Pt	30 days	60 days	90 days	1 year	2 years	3 years
BM+MPB	Pt1	99	13	136	629	1322	1194
BM	Pt2	485	300	569	1296	1083	1019
	Pt3	598	774	339	923	1964	1274
	Pt4	219	238	444	319	366	890
	Pt6	743	2375	1672	888	1075	2040
	Pt7	878	49	1106	1270	917	1179
MPB	Pt8	1019	2369	1342	1673	3216	2649
	Pt9	2767	4951	3866	7339	3738	4105
	Pt10	2781	2073	2884	3268	2445	2405
	Pt11	4014	2651	3463	3611	3286	6495

Supplementary Table 5. IS number retrieved from each WAS-GT patient in PB myeloid compartment (PB CD14+PB CD15) at each time point to perform analyses of the number of recaptured clones at early and late time points after GT. (IS= Integration Site; BM= Bone Marrow; MPB= Mobilized Peripheral Blood; PB= Peripheral Blood).

Supplementary Table 6

	BM+MPB-GT	BM-GT					MPB-GT			
Subset	Pt1	Pt2	Pt3	Pt4	Pt6	Pt7	Pt8	Pt9	Pt10	Pt11
Primitive	146	250	30	33	128	44	139	483	317	21
Erythroid	1450	306	1000	255	809	780	948	2955	988	4437
Lymphoid	5335	3940	4647	1528	6088	4765	8760	27428	15190	15707
Myeloid	4212	2084	3107	1105	3011	2161	4161	15016	9649	9033
TOTAL	11143	6580	8784	2921	10036	7750	14008	45882	26144	29198

Supplementary Table 6. Integration site number retrieved from each WAS-GT patient in Primitive (HSC+MPP), erythroid (Glycophorin+ cells), lymphoid (CD19, CD3 and CD56) and myeloid (CD14 and CD15) compartment at steady state (> 1 year after GT) to perform measurement of the hematopoietic output of engrafted HSC+MPP in BM-GT and MPB-GT patients (BM= Bone Marrow; MPB= Mobilized Peripheral Blood; GT= Gene Therapy).

Supplementary Table 7

		BM+MPB-GT	BM-GT					MPB-GT			
	Subset	Pt1	Pt2	Pt3	Pt4	Pt6	Pt7	Pt8	Pt9	Pt10	Pt11
HSPC	HSC+MPP	146	250	30	33	128	44	139	483	317	21
BM	CD14	1648	911	1538	419	1444	985	1040	3791	3754	6265
	CD15	1855	500	1049	345	1386	564	2455	4891	1765	3320
	CD19	2671	1309	2332	446	1658	1202	3487	6164	3559	5365
	CD56	1232	349	1084	409	795	1139	1629	5161	5906	7443
	GLYA	1569	307	1077	276	870	858	1059	2980	993	5324
PB	CD14	2091	2108	2091	756	1546	1760	5055	8627	1109	5400
	CD15	2852	2112	2070	819	2457	1606	4946	6555	7009	4383
	CD19	1981	2655	2272	824	2658	1762	5051	5236	3054	9560
	CD56	1009	742	939	501	1712	1584	2269	4161	1304	3751
	CD3	1251	1625	1877	500	3581	2463	4624	9170	2205	7980
TOTAL		19282	12868	16359	5328	18235	13967	31754	57219	30975	58812

Supplementary Table 7. Integration site number retrieved from each WAS-GT patient in HSPC primitive subsets, BM progenitors and PB mature populations at steady state (> 1 year after GT) to assess multilineage production of engrafted HSC+MPP in BM-GT and MPB-GT patients. (HSPC= Hematopoietic Stem/Progenitor Cells; HSC= Hematopoietic Stem Cell; MPP= Multi-Potent Progenitors; BM= Bone Marrow; PB= Peripheral Blood).
