# Supplementary material for: Hematopoietic reconstitution dynamics of mobilized- and bone marrow-derived human hematopoietic stem cells after gene therapy
Source: Nat Commun. 2023 May 27;14:3068. doi: 10.1038/s41467-023-38448-y (PMC10224916; doi:10.1038/s41467-023-38448-y)
Supplement: Supplementary file 3 — Reporting Summary [file 41467_2023_38448_MOESM3_ESM.pdf]

nature portfolio

Corresponding author(s): Aiuti Alessandro

Last updated by author(s): Apr 5, 2023

Reporting Summary

Nature Portfolio wishes to improve the reproducibility of the work that we publish. This form provides structure for consistency and transparency in reporting. For further information on Nature Portfolio policies, see our Editorial Policies and the Editorial Policy Checklist.

Statistics

For all statistical analyses, confirm that the following items are present in the figure legend, table legend, main text, or Methods section.

n/a	Confirmed	
☐	☒	The exact sample size (*n*) for each experimental group/condition, given as a discrete number and unit of measurement
☐	☒	A statement on whether measurements were taken from distinct samples or whether the same sample was measured repeatedly
☐	☒	The statistical test(s) used AND whether they are one- or two-sided<br>*Only common tests should be described solely by name; describe more complex techniques in the Methods section.*
☐	☒	A description of all covariates tested
☐	☒	A description of any assumptions or corrections, such as tests of normality and adjustment for multiple comparisons
☐	☒	A full description of the statistical parameters including central tendency (e.g. means) or other basic estimates (e.g. regression coefficient) AND variation (e.g. standard deviation) or associated estimates of uncertainty (e.g. confidence intervals)
☐	☒	For null hypothesis testing, the test statistic (e.g. *F*, *t*, *r*) with confidence intervals, effect sizes, degrees of freedom and *P* value noted<br>*Give P values as exact values whenever suitable.*
☒	☐	For Bayesian analysis, information on the choice of priors and Markov chain Monte Carlo settings
☒	☐	For hierarchical and complex designs, identification of the appropriate level for tests and full reporting of outcomes
☐	☒	Estimates of effect sizes (e.g. Cohen's *d*, Pearson's *r*), indicating how they were calculated

Our web collection on statistics for biologists contains articles on many of the points above.

Software and code

Policy information about availability of computer code

Data collection	For FACS analyses, raw data were collected through DIVA software Version 8.0.2<br>For VCN analyses, raw data were collected through QuantaSoft Version 1.7.4.0917 (Biorad).
Data analysis	For FACS analyses , raw data were analyzed with FlowJo software Version 10.5.3(BD Biosciences).<br>For VCN analyses, raw data were analyzed through QuantaSoft Version1.7.4.0917 (Biorad). Longitudinal statistical analyses on VCN data were performed using SAS 6.4<br>All the IS datasets, including the BM-GT IS previous published in Scala et al. 2018, were re-mapped with the most recent software VISPA2 (Spinozzi et al 2017). The IS analyses are described in details in the corresponding Methods section of the manuscript. Abundance estimations Chao1 and their standard errors were calculated using the R package Rcapture Version 1.4-3<br>Graphical output of all the datasets was generated thought Prism v9.1.0 software(GraphPad).

For manuscripts utilizing custom algorithms or software that are central to the research but not yet described in published literature, software must be made available to editors and reviewers. We strongly encourage code deposition in a community repository (e.g. GitHub). See the Nature Portfolio guidelines for submitting code & software for further information.

Data

Policy information about availability of data

All manuscripts must include a data availability statement. This statement should provide the following information, where applicable:

- Accession codes, unique identifiers, or web links for publicly available datasets
- A description of any restrictions on data availability
- For clinical datasets or third party data, please ensure that the statement adheres to our policy

Data supporting the current study are part of a registered clinical trial(NCT01515462). All the data generated in this study have been deposited in the San Raffaele Open Research Data Repository under accession code https://ordr.hsr.it/datasets/92gyckzm6j/draft?a=83adc1e5-79a1-4837-bf2b-9196d395454e. These data are available under restricted access for the sensitive nature of the clinical data, access can be obtained by request to the corresponding author. The not-clinical data generated in this study are also provided in the Source Data file. Additional requests should be directed to the corresponding author.

Human research participants

Policy information about studies involving human research participants and Sex and Gender in Research.

Reporting on sex and gender	Since Wiskott Aldrich syndrome(WAS) is a genetic disorders with X-linked inheritance, the cohort of WAS gene therapy treated patients is composed by male subjects.<br>We have not considered the sex as determinant in our reference dataset of bone marrow and peripheral blood cell lineage counts from pediatric healthy donors.
Population characteristics	The cohorts of pediatric HD were composed of: 7 individuals for BM evaluation(median age: 10.5 y) and 13 subjects with 1-6 years of age(median age: 4y) and 23 subjects with 6-18 years of age(median age: 12) for PB counts. WAS patients 1 to 9 were enrolled in an open-label, non-randomized, phase 1/2 clinical study8, registered with ClinicalTrials.gov(number NCT01515462) and EudraCT(number 2009-017346-32). Patients 10 to 14 were treated under expanded access program(compassionate use program or hospital exemption). Detailed patients' characteristics are reported in Table 1.
Recruitment	In order to analyze the groups of BM-GT vs. MPB-GT patients with the same range of age, we included in this study all the pediatric GT patients treated since 2010 up to 2018, with the exception of one patient who shows an undetectable CD90 marker expression, likely due to modification in the protein conformation(currently under investigation). In particular, the ranges of age at treatment were from 1.9 and 5.9 years (median age:1.9) for the BM-GT group and from 1.4 to 14.4 years (median age: 10.3) for MPB-GT patients. The data cutoff was chosen to evaluate all the available patients up to 2 year-follow up. The choice of the source was based on the evaluation of different clinical factors including age and weight of the patient, estimated yield of CD34+ cells (according to the content of bone marrow CD34+ cells evaluated by previous bone marrow aspiration, performed at screening) and feasibility of the procedure. Moreover, a mixture of MPB and BM CD34+ cells was foreseen if required to obtain a sufficient cells dose. In particular, Pt1 required administration of cell drug product from both cell sources for reaching optimal cell dose. For Pt#2-7 (BM-GT) and for Pt#8-9 (MPB-GT) the source was chosen due to the age of the patients. Indeed, BM harvest was the preferred choice for younger pediatric subjects at the time of the procedure. With the introduction of the Plerixafor in the mobilization protocol, the improvement of the clinical management of the leukapheresis procedure as well as the CD34+ cell yield obtained in patients Pt#8-9, the remaining patients treated in the expanded access program (Pt#10-14) were treated with MPB CD34+ cells. All the described patients are alive, clinically well and no adverse event related to the product has been observed to date.
Ethics oversight	Pediatric HD and WAS samples were analyzed after parents signed informed consent approved by the San Raffaele Scientific Institute's Ethics Committee(TIGET06 and TIGET09) at IRCCS Ospedale San Raffaele.

Note that full information on the approval of the study protocol must also be provided in the manuscript.

Field-specific reporting

Please select the one below that is the best fit for your research. If you are not sure, read the appropriate sections before making your selection.

☒ Life sciences ☐ Behavioural & social sciences ☐ Ecological, evolutionary & environmental sciences

For a reference copy of the document with all sections, see nature.com/documents/nr-reporting-summary-flat.pdf

Life sciences study design

All studies must disclose on these points even when the disclosure is negative.

Sample size	For phenotypic characterization of WAS patients we collected all in vivo biological material available during years 2010-2018 (after informed consent from parent's patients) . We aimed at analyzing for all the patients pre- and post-treatment mature lineages counts and HSPC composition and absolute numbers. We aimed at measuring VCN in mature lineages for all the patients at all available time points. For IS clonal tracking studies we aimed at isolating IS from all the patients included in the research study.<br>For the comparison with Pediatric healthy donors we aimed at collecting at least 10 different subjects and at least 5 different donors/source

(BM or PB). These numbers were chosen to match the number of the GT patients included in our research study.
For in vitro and in vivo assay to compare primitive HSPC from BM or MPB isolated from adult HD, we aimed at analyzing at least 3 different donors/source. Moreover, we aimed at transplanting at least 3 mice for donor and for each cell dose, reaching a minimal number of 9 transplanted mice/experimental group. These numbers allow performing non-parametric statitical analyses to compare different group.

Data exclusions

All available healthy donors' and patients' samples are reported in the manuscript.Technically validated results were always included to the analyses and we did not apply any exclusion criteria for outliers.

Replication

For phenotypic characterization of HSPC population, Rainbow beads (RB) calibration was performed during the set up of the instrumentation for FACS analyses. RB acquisition was performed before each sample acquisition in order to achieve reproducible instrument setting among different experiments. All the patients' and healthy donors' samples were acquired independently. We analyzed all the available samples.
For VCN evaluation, ddPCR was validated for reproducibility. We run each sample in triplicate and values are reported as mean of the three triplicates.
For in vitro assay, each sample was run in duplicate and values are reported as mean of the two duplicates.
For in vivo assay, since we retreived different amount of primitive HSPC from BM and MPB CD34+ cells (due to differential composition of the two sources), we transplanted for each BM donors all the possible mice with 10K cell dose, while for MPB donors we transplanted the same number of mice with 10K cell dose and 45K cell dose.

VCN evaluations as well as in vitro and in vivo experiments were performed independently and all the attempt for replication were successfull.

Randomization

For the in vivo experiments, mice were randomly assigned to distinct experimental groups.

Blinding

The main focus of the work was to compare the effect of HSPC source on two cohorts of GT treated patients. For this reason blinding was not applicable to our study.

Reporting for specific materials, systems and methods

We require information from authors about some types of materials, experimental systems and methods used in many studies. Here, indicate whether each material, system or method listed is relevant to your study. If you are not sure if a list item applies to your research, read the appropriate section before selecting a response.

Materials & experimental systems

n/a	Involved in the study	
☐	☒	Antibodies
☒	☐	Eukaryotic cell lines
☒	☐	Palaeontology and archaeology
☐	☒	Animals and other organisms
☐	☒	Clinical data
☒	☐	Dual use research of concern

Methods

n/a	Involved in the study	
☒	☐	ChIP-seq
☐	☒	Flow cytometry
☒	☐	MRI-based neuroimaging

Antibodies

Antibodies used

Mouse anti-human CD3-BV605, Clone OKT3 (Biolegend, 317322), Dilution:1:50
Mouse anti-human CD56-PC5, Clone 5.1H11 (Biolegend, 362516), Dilution:1:50
Mouse anti-human CD14-BV510, Clone M5E2 (Biolegend, 301842), Dilution:1:50
Mouse anti-human CD33-BB515, Clone WM53 (BD Biosciences, 564588), Dilution:1:50
Mouse anti-human CD41/CD61-PC7, Clone A2A9/6 (Biolegend, 359812), Dilution:1:50
Mouse anti-human CD66b-BB515, Clone G10F5 (BD Biosciences, 564679), Dilution:1:50
Mouse anti-human CD7-BB700, Clone M-T701 (BD Biosciences, 566488), Dilution:1:50
Mouse anti-human CD45-BUV395, Clone HI30 (BD Biosciences, 563792), Dilution:1:33
Mouse anti-human CD38-BUV737, Clone HB7 (BD Biosciences, 612824), Dilution:1:33
Mouse anti-human CD90-APC, Clone 5E10 (BD Biosciences, 559869), Dilution:1:33
Mouse anti-human CD135-PE, Clone BV10A4H2 (Biolegend, 313306), Dilution:1:33
Mouse anti-human CD184(CXCR4)-PE, Clone 12G5 (Biolegend, 306506),Dilution:1:50
Mouse anti-human CD11c-BV650, Clone B-ly6 (BD Biosciences, 563404), Dilution:1:20
Mouse anti-human CD10-BV786, Clone HI10a (BD Biosciences, 564960), Dilution:1:20
Mouse anti-human CD34-BV421, Clone 561 (Biolegend, 343610), Dilution:1:20
Mouse anti-human CD45RA-APCH7, Clone HI100 (Biolegend, 304128), Dilution:1:20
Mouse anti-human CD71-BV711, Clone M-A712 (BD Biosciences, 563767), Dilution:1:20
Mouse anti-human CD19-APCR700, Clone SJ25C1 (BD Biosciences, 659121), Dilution:1:20
Mouse anti-human Lineage cocktail (anti-CD3/CD14/ CD16/CD19/CD20/CD56)-BV510, Clones OKT3, M5E2, 3G8, HIB19, 2H7, HCD56 (Biolegend, 348807), Dilution:1:10
Mouse anti-human CD15-BV510, Clone W6D3 (Biolegend, 323028), Dilution:1:50
Mouse anti-human CD34-PB, Clone 581 (Biolegend, 343512), Dilution:1:20
Mouse anti-human CD38-PC5, Clone HIT2 (Biolegend, 303508), Dilution: 1:50
Mouse anti-human CD10-PC7, Clone HI10a (Biolegend, 312214), Dilution:1:33
Mouse anti-human CD7-APCR700, Clone M-T701 (BD Biosciences, 659124), Dilution:1:20
Mouse anti-human CD235a-PE, Clone GA-R2 (HIR2) (BD Biosciences, 561051), Dilution:1:100

Mouse anti-human CD1a-APC, Clone HI149 (BD Biosciences, 561755), Dilution:1:33
Mouse anti-human CD5-BUV737, Clone UCHT2 (BD Biosciences, 612842),Dilution:1:33
Mouse anti-human CD42b-BV786, Clone HIP1 (BD Biosciences, 740976), Dilution: 1:20
Mouse anti-human CD41-PC7, Clone HIP8 (Biolegend, 303718), Dilution:1:50
Mouse anti-human CD10-BV510, Clone HI10a (Biolegend, 312219), Dilution:1:100
Mouse anti-human CD15-APCfire750, Clone W6D3 (Biolegend, 323041), Dilution:1:50

Validation

All the antibodies were purchased from Biolegend and BD Bioscience and they are well characterized and validated by providers.
Mouse anti-human CD3-BV605, Verified Reactivity: Human; Application: Flow cytometric analysis of antibody surface-stained cells.
Mouse anti-human CD56-PC5, Verified Reactivity: Human; Application: Flow cytometric analysis of antibody surface-stained cells.
Mouse anti-human CD14-BV510, Verified Reactivity: Human, Cynomolgus, Rhesus; Application: Flow cytometric analysis of antibody surface-stained cells.
Mouse anti-human CD33-BB515, Verified Reactivity: Human (QC Testing); Application: Flow cytometry (Routinely Tested)
Mouse anti-human CD41/CD61-PC7, Verified Reactivity: Human; Application: Flow cytometric analysis of antibody surface-stained cells.
Mouse anti-human CD66b-BB515, Verified Reactivity: Human (QC Testing); Application: Flow cytometry (Routinely Tested)
Mouse anti-human CD7-BB700, Verified Reactivity: Human (QC Testing) , Rhesus, Cynomolgus, Baboon (Reported); Application: Flow cytometry (Routinely Tested)
Mouse anti-human CD45-BUV395, Verified Reactivity: Human (QC Testing); Application: Flow cytometry (Routinely Tested)
Mouse anti-human CD38-BUV737, Verified Reactivity: Human (QC Testing); Application: Flow cytometry (Routinely Tested)
Mouse anti-human CD90-APC, Verified Reactivity: Human (QC Testing), Rhesus, Cynomolgus, Baboon, Pig, Dog (Tested in Development); Application: Flow cytometry (Routinely Tested)
Mouse anti-human CD135-PE, Verified Reactivity: Human; Application: Flow cytometric analysis of antibody surface-stained cells.
Mouse anti-human CD184(CXCR4)-PE, Verified Reactivity: Human, Cynomolgus, Rhesus; Application: Flow cytometric analysis of antibody surface-stained cells.
Mouse anti-human CD11c-BV650, Verified Reactivity: Human (QC Testing); Application: Flow cytometry (Routinely Tested)
Mouse anti-human CD10-BV786, Verified Reactivity: Human (QC Testing), Rhesus, Cynomolgus, Baboon (Tested in Development); Application: Flow cytometry (Routinely Tested)
Mouse anti-human CD34-BV421, Verified Reactivity: Human; Application: Flow cytometric analysis of antibody surface-stained cells.
Mouse anti-human CD45RA-APCH7, Verified Reactivity: Human; Reported Reactivity: Chimpanzee; Application: Flow cytometric analysis of antibody surface-stained cells.
Mouse anti-human CD71-BV711, Verified Reactivity: Human (QC Testing); Application: Flow cytometry (Routinely Tested)
Mouse anti-human CD19-APCR700, Verified Reactivity: Human; Application: Flow cytometry
Mouse anti-human Lineage cocktail (anti-CD3/CD14/ CD16/CD19/CD20/CD56)-BV510, Verified Reactivity: Human; Application: Flow cytometric analysis of antibody surface-stained cells.
Mouse anti-human CD15-BV510, Verified Reactivity: Human; Application: Flow cytometric analysis of antibody surface-stained cells.
Mouse anti-human CD34-PB, Verified Reactivity: Human; Reported Reactivity: Cynomolgus; Application: Flow cytometric analysis of antibody surface-stained cells.
Mouse anti-human CD38-PC5, Verified Reactivity: Human; Reported Reactivity: Chimpanzee, Horse, Cow; Application: Flow cytometric analysis of antibody surface-stained cells.
Mouse anti-human CD10-PC7, Verified Reactivity: Human, Cynomolgus, Rhesus; Reported Reactivity: African Green, Baboon, Capuchin monkey, Chimpanzee; Application: Flow cytometric analysis of antibody surface-stained cells.
Mouse anti-human CD7-APCR700, Verified Reactivity: Human; Application: Flow cytometry
Mouse anti-human CD235a-PE, Verified Reactivity: Human (QC Testing); Application: Flow cytometry (Routinely Tested)
Mouse anti-human CD1a-APC, Verified Reactivity: Human (QC Testing); Application: Flow cytometry (Routinely Tested)
Mouse anti-human CD5-BUV737, Verified Reactivity: Human (QC Testing); Application: Flow cytometry (Routinely Tested)
Mouse anti-human CD42b-BV786, Verified Reactivity: Human (Tested in Development); Application: Flow cytometry (Qualified)
Mouse anti-human CD41-PC7, Verified Reactivity: Human; Reported Reactivity: African Green, Baboon, Capuchin Monkey, Cynomolgus, Rhesus; Application: Flow cytometric analysis of antibody surface-stained cells.
Mouse anti-human CD10-BV510, Verified Reactivity: Human, Cynomolgus, Rhesus; Reported Reactivity: African Green, Baboon, Capuchin monkey, Chimpanzee; Application: Flow cytometric analysis of antibody surface-stained cells.
Mouse anti-human CD15-APCfire750, Verified Reactivity: Human; Application: Flow cytometric analysis of antibody surface-stained cells.

Animals and other research organisms

Policy information about studies involving animals; ARRIVE guidelines recommended for reporting animal research, and Sex and Gender in Research

Laboratory animals

6-7 weeks old NOD.Cg-KitW-41J Prkdcscid Il2rgtm1Wjl/WaskJ(NSGW41, stock #026497) mice were purchased from the Jackson Laboratory and transplanted for the in vivo assay.All animals were maintained in the SPF animal facility at IRCCS Ospedale San Raffaele with no more than 5 animals per cage (cage covered with filter, sterile air ventilation) at a temperature in the range 20° to 24°C and a relative humidity of 45-65%; at approximately one cycle of light (12 hours light and 12 hours dark) with ad libitum food and water. The type of diet used was VRF1(P), a GLP certificated rat and mouse breeding diet, containing elevated levels of heat labile vitamins, which make it suitable for autoclaving and for animals with high vitamin requirements (i.e. SPF/Germ free). The handling of the animals was performed by trained personnel, with the aim of minimizing the degree of stress and suffering and the period of constraint. The degree of well-being and the clinical conditions of the animals were assessed by daily post- treatment or transplantation observation. During detailed clinical observation the following parameters were evaluated: loss of body weight, posture, activity level, fur and skin appearance, breathing and reaction to manipulation. All animals were euthanized by CO2 inhalation as described in our specific authorized research project and carried out following the AVMA Guidelines for the Euthanasia of Animals: 2020 Edition.

Wild animals

The study did not include wild animals.

Reporting on sex	Sex was not considered in the study design
Field-collected samples	This study did not involve samples collected form the field
Ethics oversight	Mouse studies were conducted according to protocols approved by the San Raffaele Scientific Institute and the Italian Ministry of Health(IACUC, #1091)

Note that full information on the approval of the study protocol must also be provided in the manuscript.

Clinical data

Policy information about clinical studies

All manuscripts should comply with the ICMJE guidelines for publication of clinical research and a completed CONSORT checklist must be included with all submissions.

Clinical trial registration	WAS patients 1 to 9 were enrolled in an open-label, non-randomized, phase 1/2 clinical study8, registered with ClinicalTrials.gov(number NCT01515462) and EudraCT(number 2009-017346-32). Patients 10 to 14 were treated under expanded access program(compassionate use program or hospital exemption).
Study protocol	The clinical trial study protocol can be found in the uploades "Related Manuscript Files"
Data collection	For clinical data, source data include all the documents related to the patient during the trial or patient's treatment under expanded access (infused CD34+ cell dose, VCN and % of transduction of the infused cell dose, blood cell counts, requirement for platelet transfusion, VCN in PB and BM subpopulation, VCN and % of transduction on BM CFC); these data were collected at Ospedale San Raffaele (Milan, Italy) and entered in the respective case report form and monitored according to the clinical trial/expanded access protocol and local guidelines. Clinical data were collected at IRCCS Ospedale San Raffaele starting from 2010. All the other exploratory research data (flow cytometry and integration site analyses) were collected at San Raffaele Telethon Institute for Gene Therapy (from 2010 to 2021) and they were analyzed and stored in a dedicated database.
Outcomes	This manuscript does not report on the primary and secondary outcome of the clinical trial which have been reported as interim analyses in the paper by Ferrua et al. , Lancet Haematology 2019. The comparison between MPB and BM was not pre-specified in the study protocol and it has been performed as additional exploratory analyses for the purpose of this publication and approved by the San Raffaele Scientific Institute's Ethics Committee at IRCCS Ospedale San Raffaele.

Flow Cytometry

Plots

Confirm that:

☒ The axis labels state the marker and fluorochrome used (e.g. CD4-FITC).

☒ The axis scales are clearly visible. Include numbers along axes only for bottom left plot of group (a 'group' is an analysis of identical markers).

☒ All plots are contour plots with outliers or pseudocolor plots.

☒ A numerical value for number of cells or percentage (with statistics) is provided.

Methodology

Sample preparation	Identification and quantification of HSPC subpopulations from BM-GT and Pt1 were previously performed (Scala et al 2018). MPB-GT patients' PB and BM samples as well as the BM of transplanted mice were analyzed using Whole Blood Dissection(WBD) cytometry assay (Basso-Ricci et al 2017). Briefly, after red blood cell lysis, BM and PB samples were labeled with fluorescent antibodies against CD3, CD56, CD14, CD61/41, CD135, CD34, CD45RA (Biolegend) and CD33, CD66b, CD38, CD45, CD90, CD10, CD11c, CD19, CD7 and CD71 (BD Biosciences). Titration assays were performed to assess the best antibody concentration. After surface marking, the cells were incubated with PI (Biolegend) to stain dead cells. Absolute cell quantification was performed by adding Flowcount beads(BD Bioscience) to samples before WBD procedure.
Instrument	All stained samples were acquired through BD LSR-Fortessa or BD Symphony A5(BD Bioscience) cytofluorimeters after Rainbow beads(Spherotech) calibration.
Software	Raw data were collected through DIVA software and analyzed with FlowJo software Version 10.5.3(BD Biosciences).
Cell population abundance	Sorted cell reached a purity ranging between 92% and 99%. Due to the low amount of starting materials we run in parallel an healthy donor sample to check the efficiency and purity of our sorting by acquiring sorted subpopulations at the analyzer. Moreover, the double-sorting strategy allows an internal control of purity of our sorted populations.
Gating strategy	The gating strategy for the identification of HSPC subsets was described in Supplementary Fig 1.<br>Briefly<br>1. Create a dot plot FSC-H/FSC-A to identify single cells (G1).<br>2. Create on G1 a dot plot SSC-A/FSC-A to identify the cells-size population (G2).<br>3. Create on G2 a dot plot PI-A/FSC-A to select live cells (low PI-A) (G3). From now on each gate has to be set according to its respective FMO control.

5. Create on G3 a dot plot SSC-A/BUV395-A to evaluate the expression of CD45 maker. Create a gate on CD45 negative (G4) and CD45 positive events (G5).
4. Create on G5 a dot plot BUV737-A/BV421-A to evaluate the expression of CD38 and CD34 markers and create a gate on negative CD34 (G6) and on positive CD34 events (G7).
6. Create on G7 a dot plot SSC-A/BB515-A to evaluate the expression of CD33/CD66b markers. Create a gate on negative (G8; not-myeloid progenitors) and positive events (G9; myeloid progenitors).
7. Create on G9 a dot plot SSC-A/BV510-A to evaluate the expression of CD14 marker and cell granularity (SSC-A). Create a gate on CD14+ (G10), on high granularity events (G11) and on negative events (G12).
8. Create on G11 a dot plot BV786-A/BV650-A to evaluate the expression of CD10 and CD11c markers and create a gate on double positive events (G13) and on remaining events (G14).
9. Create on G12 a dot plot SSC-A/BV650-A to evaluate the expression of CD11c marker and create a gate on positive (G15) and negative events (G16).
10. Create on G16 a dot plot BUV737-A/BV421-A to evaluate the expression of CD38 and CD34 markers. Create a gate on CD34 positive events (G17; myeloid-committed progenitors). Check that all the events in this dot plot follow into the G17 gate.
11. Create on G8 a dot plot SSC-A/BV605-A to evaluate the expression of CD3 marker and create a gate on positive (G18) and negative events (G19).
12. Create on G18 a dot plot SSC-A/PE-Cy5-A to evaluate the expression of CD56 and create a gate on positive (G20) and negative events (G21).
13. Create on G19 a dot plot SSC-A/APC-R700-A to evaluate the expression of CD19 marker and create a gate on positive (G22) and negative (G23) events.
14. Create on G22 a dot plot BV786-A/BV421-A to evaluate the expression of CD10 and CD34 markers and create a gate on double positive (G24; Pro-B), and CD34+only (G25) events. Check that all the events in this dot plot follow into G24 or G25 gates.
15. Create on G23 a dot plot SSC-A/PE-Cy5-A to evaluate the expression of CD56 marker and create a gate on positive (G26) and negative (G27) events.
16. Create on G27 a dot plot BUV737-A/BV421-A to evaluate the expression of CD38 and CD34 markers and create a gate on CD34+ (G28, LIN- progenitors). Check that all the events in this dot plot follow into the G28 gate.
17. Create on merged G17+G28 a dot plot BUV737-A/BV421-A to evaluate the expression of CD38 and CD34 markers. All the events should be CD34+. Create a gate on CD38+ (G29) and CD38- (G30) events.
18. Create on G30 a dot plot APC-A/APC-Cy7-A to evaluate the expression of CD90 and CD45RA markers and create a gate on CD90+only (G31; HSC), double negative (G32; MPP) and CD45RA+ (G33; MLP) events.
19. Create on G29 a dot plot SSC-A/BB700 to evaluate the expression of CD7 marker and create a gate on CD7+ (G34; ETP) and CD7- (G35) events.
20. Create on G35 a dot plot BV786-A/APC-Cy7-A to evaluate the expression of CD10 and CD45RA markers and create a gate on CD10+ (G36; Pre-B/NK) and CD10- (G37) events.
21. Create on G37 a dot plot PE-A/APC-Cy7-A to evaluate the expression of CD135 and CD45RA markers and create a gate on CD135+ only (G38; CMP), double positive (G39; GMP) and double negative (G40; MEP) events.

☒ Tick this box to confirm that a figure exemplifying the gating strategy is provided in the Supplementary Information.
